# Dramatic improvement of strain hardening and ductility to 95% in highly-deformable high-strength duplex lightweight steels

**DOI:** 10.1038/s41598-017-02183-4

**Published:** 2017-05-16

**Authors:** Seok Su Sohn, Hyejin Song, Jai-Hyun Kwak, Sunghak Lee

**Affiliations:** 1 Center for Advanced Aerospace Materials, Pohang University of Science and Technology, Pohang, 790-784 Korea; 2 Sheet Products & Process Research Group, Technical Research Laboratories, POSCO, Kwangyang, 545-090 Korea

## Abstract

Ferrite + austenite duplex lightweight steels have been actively developed by adding low-density Al for overcoming a limitation of stiffness deterioration by a traditional approach to obtain a weight reduction. Multiple-stage deformation mechanism in lightweight steels, *i.e*., simultaneous formation of deformation-induced martensite and deformation twin and additional plasticity by twinning, has been nominated as an attractive strategy, but shows a steady flow behavior with early plastic instability. Here, we present a newly designed Fe-0.3C-9Mn-5Al steel in order to obtain an optimal level of stability of austenite and a resultant outstanding combination of tensile strength and ductility, *e.g*., 874 MPa and 72%, together with sufficiently high strain hardening. These enhanced properties are attributed to the decreased austenite stability by controlling the austenite size and alloying partitioning due to variation in austenite fraction inside duplex microstructures. The present work gives a promise for structural applications requiring both reduced specific weight and remarkable deformability.

## Introduction

Effective control of global warming and greenhouse gas reduction have become key issues in automotive industries^1–5^. A conventional approach for vehicle’s weight reduction is trimming down the size of high-strength steel components by appropriately designing high-strength microstructures, but has a limitation due to deterioration of stiffness and structural rigidity^6, 7^. As a compromising way, new advanced automotive steels, *e.g*., lightweight steels, have been actively developed by simply adding low-density Al^8–19^. As well as their design toward the higher strength, the higher elongation is needed to meet tough requirements of enhanced sheet formability in automotive structural parts. On the contrary to conventional strength *vs*. ductility relationship, in order to achieve a maximization of both strength and ductility, powerful deformation mechanisms of TRansformation Induced Plasticity (TRIP) and TWinning Induced Plasticity (TWIP) have been presented as desirable ones^20–25^. We reported excellent tensile properties of tensile strength of 734 MPa and elongation of 77% in an Fe-0.3C-8.5Mn-5.6Al steel^26^. This is because the TWIP mechanism is working together with the TRIP mechanism, and because deformation twins and α’-martensite are independently formed inside even one austenite grain.

Key ideas of (TRIP + TWIP) mechanisms are based on the distribution of stored energy by TWIP as well as TRIP, and on the maximization of delayed necking induced by additional plasticity due to TWIP. They contributes the ductility enhancement, but the TRIP rate is too low, while the TWIP does not greatly contribute the strength enhancement, thereby showing a steady flow behavior in the strain range of 30~77%. This steady flow, which can be regarded as an inhomogeneous localized deformation including necking and shear banding, becomes a major drawback for structural applications, although it improves a combination of strength and ductility. Plastically unstable materials often create environments, under which even small defects promote the inhomogeneous localized deformation, which can readily lead to the deterioration of deformability, fracture resistance, and energy absorption capability^27, 28^. Delaying plastic instability to higher strain levels is desirable for toughness enhancement as well as forming processes, and has become a real concern in structural materials design.

Since (TRIP + TWIP) mechanisms are sensitively varied with stability or SFE of austenite^26, 29–32^, tensile properties can be improved by obtaining an optimal range of stability or SFE. Especially for the increase in strain hardening rate, more active TRIP mechanisms are generally needed. Here, we optimize the stability of austenite by controlling the austenite size and alloying partitioning due to variation in austenite fraction inside duplex microstructures, instead of simple alloy designing based on martensite start (Ms) temperature calculation. The austenite stability is indirectly decreased by the increased austenite fraction due to increased Mn and decreased Al contents, instead of the direct decrease in austenite stability due to the decreased C content. Since it is sensitively working for the strain hardening effect even when small amounts of alloying elements are varied, a remarkable improvement of strain hardening without plastic instability as well as high yield strength can also be achieved by further optimization of austenite size and fraction.

## Results and Discussion

### Alloy design concept

The Fe-0.3C-8.5Mn-5.6Al steel, which is referred to as a previous steel for convenience, shows the tensile strength of 734 MPa and elongation of 77%, but its tensile behavior shows a steady stress flow after the rapidly increased strain hardening^26^. This is attributed to the very low TRIP rate, although both TRIP and TWIP are working above the strain of 30%. Since the active TRIP is required for the high strain hardening rate, the present alloy design mainly focuses on the achievement of higher strain hardening effect by decreasing the austenite stability, while the excellent ductility is maintained. The steel composition and austenite grain size are main parameters affecting austenite stability. In the presently modified Fe-0.3C-9Mn-5Al lightweight steel composition, C and Mn raise the austenite stability, whereas Al reduces it. When C and Mn contents are reduced, the austenite stability is easily expected to be decreased, but can be increased by the partitioning in austenite because of the reduction in austenite volume fraction. In the present alloy design concept, thus, the austenite stability is reduced by the increase in Mn content and decrease in Al content, together with the decrease in average C content in austenite. Since the austenite grain size can be appropriately controlled by annealing treatments, its effect will be discussed later.

**Figure 1:**
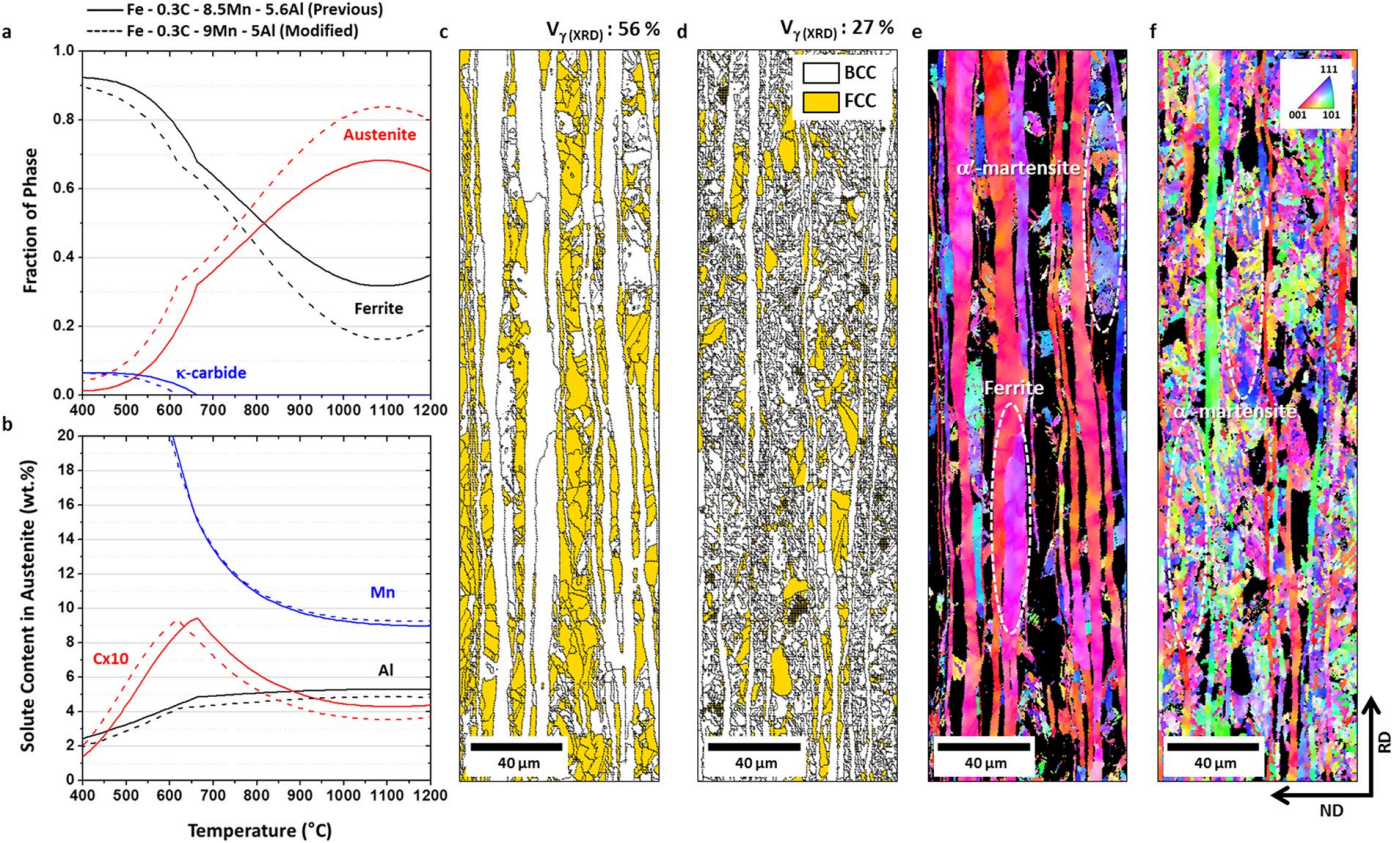
Phase calculations and actual hot-rolled microstructures of previous and modified steels. (**a**) High-temperature equilibrium phase distribution. (**b**) Solute contents in the austenite phase calculated by using ThermoCalc^28–30^. (**c–f**) EBSD phase and inverse pole figure (IPF) maps. All the EBSD maps are obtained from hot-rolled plates. Both steels have (ferrite + austenite) duplex microstructures in a banded shape elongated along the rolling direction.

Figure 1(a,b) shows fractions of phases and solute contents in austenite calculated by a commercial program, Thermocalc^33–35^, for the previous and modified steels. In Fig. 1(b), the C content is ten-times expanded because it is much lower than the Mn and Al contents. When small amounts (*e.g*., about 0.5 wt.%) of Mn and Al are varied, the austenite fraction is varied by about 15% at 1100 °C (Fig. 1(a)). In the austenite, the C and Al contents decrease, while the Mn content is almost retained (Fig. 1(b)). In order to examine effects of variation in alloying contents, calculated fractions, EBSD phase and inverse pole figure (IPF) maps of the hot-rolled plates of the previous and modified steels are shown in Fig. 1(c–f). The steels have duplex microstructures of ferrite and austenite in a banded shape elongated along the rolling direction (Fig. 1(c,d)). The austenite fractions are 56% and 27% in the previous and modified steels, respectively. According to IPF maps, BCC phases are classified into elongated ferrite and α’-martensite formed inside austenite bands during the cooling, as indicated by white dashed ellipses in Fig. 1(e,f). The volume fraction of ferrite is considerably lower in the modified steel than in the previous steel, while that of α’-martensite is higher. As the average C content inside the austenite decreases and austenite fraction increases during the reheating and hot-rolling processes in the range of 1200 °C~900 °C (Fig. 1(a,b)), the volume fraction of martensite increases in the modified steel (Fig. 1(f)). The actually measured Mn and Al contents are 9.62Mn-5.05Al and 10.55Mn-4.48Al (in wt.%) in the previous and modified steels, respectively, but are positioned in the range of 770 °C~920 °C, as shown in the thermodynamically diagram (Fig. 1(b)). These results imply that the austenite stability can be sufficiently reduced by small amounts (about 0.5 wt.%) of Mn and Al, like in the modified steel.

### Improvement of strain hardening in modified duplex lightweight steel

**Figure 2:**
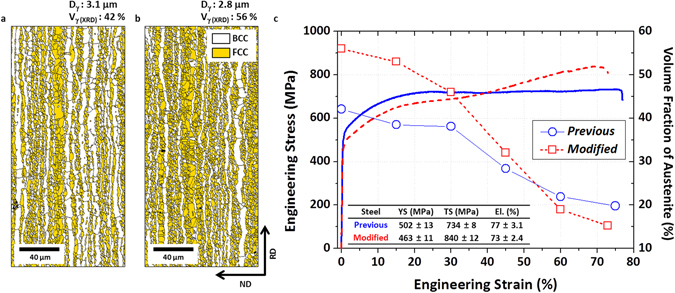
Microstructures and tensile properties of previous and modified steels. (**a,b**) EBSD phase map. (**c**) room temperature tensile stress-strain curves. The austenite grain sizes are similar (about 3 μm), but the austenite fraction is higher in the modified steel (56%). The previous steel shows a steady stress flow behavior after the strain of 30% without a large strain hardening, whereas the modified steel shows a continuous strain hardening after the strain of 25%. Thus, the modified steel has the higher tensile strength (840 MPa) than the previous steel, while the elongation is almost same. Both steels were cold-rolled with reduction ratio of 66%, and were annealed at 900 °C for 30 min.

Figure 2(a,b) shows EBSD phase maps of the previous and modified steels after the 66%-cold-rolling followed by annealing at 900 °C for 30 min. The austenite grain sizes are similar (about 3 μm), but the austenite fraction is higher in the modified steel (56%). Figure 2(c) shows engineering stress-strain curves and volume fraction of austenite. The previous steel shows a steady stress flow behavior after the strain of 30% without a large strain hardening, whereas the modified steel shows a continuous strain hardening after the strain of 25%. Thus, the modified steel has the higher tensile strength (840 MPa) than the previous steel, while the elongation is almost same. With respect to TRIP, the austenite fraction of the modified steel is higher before the tensile deformation than that of the previous steel, decreases rather rapidly, and becomes lower after the strain of 0.6, which indicates the active TRIP due to the decreased austenite stability. This result implies that a concept of indirectly decreased austenite stability by the increased austenite fraction, instead of by the decreased C content, is favorably working in the modified steel, and that the strain hardening effect is sensitively influenced by a small amount of alloying elements.

### Novel tensile properties by controlling austenite fraction and grain size

As aforementioned above, the appropriate control of alloying elements leads to more excellent tensile properties in the modified steel than in the previous steel. The austenite grain size as well as alloying element acts as major parameters affecting the austenite stability. In order to investigate effects of composition and grain size of austenite on stability in the modified steel, the annealing at 850 °C, 900 °C, and 950 °C for 5 min was conducted, and the annealed steels are referred to as ‘A850’, ‘A900’, and ‘A950’, respectively. According to XRD and EBSD phase map analyses, the volume fraction and size of austenite grain are 46% and 1.7 μm, respectively, in the A850 steel, and increase to 54% and 2.1 μm, and 60% and 2.6 μm, respectively, in the A900 and A950 steels. When the 5-min-annealed A900 steel is compared with the 30-min-annealed steel (Fig. 2(b)), both austenite grain size and fraction are slightly smaller in the A900 steel (2.1 μm *vs* 2.8 μm and 54% *vs* 56%, respectively).

**Figure 3:**
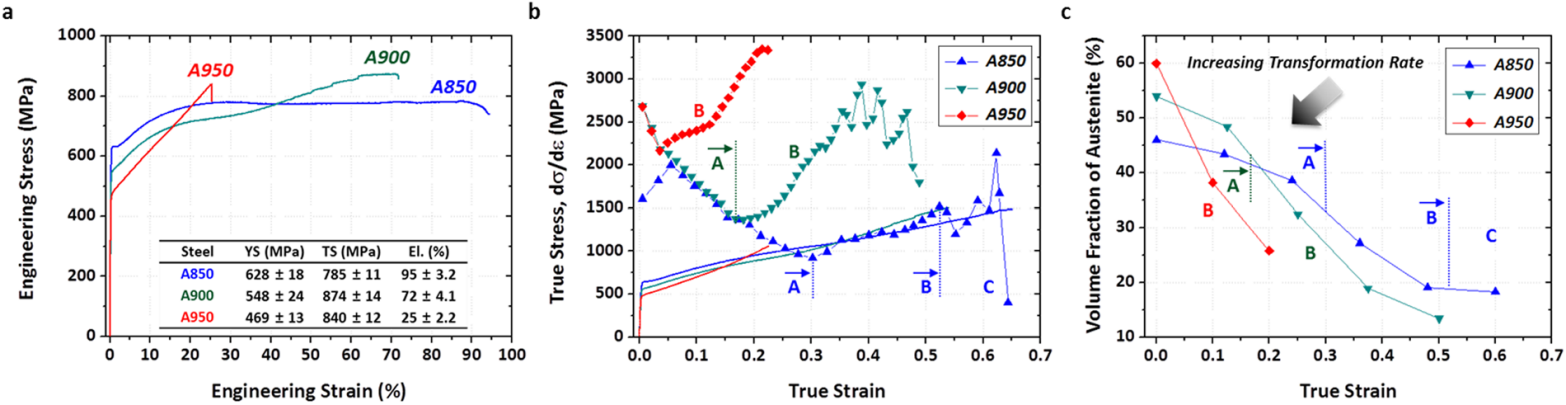
Tensile properties, strain hardening rate, and martensitic transformation rate of the annealed steels. (**a**) Room temperature engineering tensile stress-strain curves. (**b**) True stress-strain and strain hardening rate (dσ/dε) curves. In the A850 steel, the stress flow becomes steady after the rapidly increased strain hardening, while the elongation reaches 95%. The plastic instability starts to occur at the true strain of 0.25, and continues during the steady flow. In the A900 steel, the tensile strength increases to 874 MPa as the strain hardening rate increases, while the yield strength and elongation decrease. The plastic instability does not occur until the failure because the strain hardening rate is considerably higher than the true stress. (**c**) Volume fraction of austenite as a function of true strain. The volume fraction of austenite decreases with increasing true strain because of TRIP. The transformation behavior is classified into several stages, *e.g*., slowly decreasing stage (Stage A), rapidly decreasing stage (Stage B), and steady stage (Stage C).

Figure 3(a) shows engineering stress-strain curves, from which tensile properties are summarized inside the figure. In the A850 steel, the yield strength, tensile strength, and elongation are 628 MPa, 785 MPa, and 95%, respectively. This elongation is quite outstanding one, which has not been reported in previous studies on duplex lightweight steels^24, 29, 36, 37^. The stress flow becomes steady after the rapidly increased strain hardening. This indicates that its strength and elongation are improved over the previous steel, although the steady flow behavior is similarly shown. With increasing annealing temperature to 900 °C, the tensile strength increases to 874 MPa as the strain hardening rate increases, while the yield strength and elongation decrease. In the A950 steel, the elongation abruptly reduces down to about 25%, although the tensile strength slightly increases to 840 MPa.

Figure 3(b) shows true stress-strain curves and strain hardening rate (dσ/dε) curves of the annealed steels. The true stress-strain curve of the A850 steel shows a continuously increasing strain hardening behavior, which is different from the steady stress flow behavior of the engineering stress-strain curve, and the true tensile strength reaches 1479 MPa. The A900 steel shows the highest true tensile strength (1496 MPa) among the three annealed steels. In the A850 steel, the plastic instability starts to occur at the true strain of 0.25, where both stress and strain hardening rate are same at 1000 MPa, and continues during the steady flow. This implies that a drawback of the previous steel, *i.e*., steady flow, remains to be addressed in the A850 steel in spite of the dramatic improvement of tensile elongation to 95%. In the 900 steel, on the other hand, the plastic instability does not occur until the failure because the strain hardening rate is considerably higher than the true stress.

### Deformation mechanisms affecting the transition in strain hardening behavior

The strain hardening rate curves display a multiple-stage strain hardening behavior in Fig. 3(b). Based on the multiple-stage strain hardening behavior in the previous steel^26^, the present deformation stages are defined to be active slip mechanism as well as occurrence of a small amount of TRIP in the Stage A, beginning of active TRIP and TWIP in the Stage B, and active TWIP and a small amount of TRIP in the Stage C. The strain hardening rate continuously decreases in the Stage A, and then increases from the Stage B. In the Stage C whose austenite fraction is hardly varied, the hardening rate tends to slightly increase with some serrated flows. The overall strain hardening rate increases with increasing annealing temperature.

Volume fractions of austenite as a function of true strain are shown in Fig. 3(c). The volume fraction of austenite decreases with increasing true strain in the three annealed steels, which implies the occurrence of TRIP. The transformation behavior is classified into several stages, *e.g*., slowly decreasing stage (Stage A), rapidly decreasing stage (Stage B), and steady stage (Stage C), as marked by ‘A’ through ‘C’ on each curve in Fig. 3(c). In the A850 steel, the Stage A is continued to the true strain of 0.3, and then the Stage B and C appear consecutively. As the annealing temperature increases, the Stage A is shortened, the Stage B is extended, and the Stage C tends to disappear. That is, the critical strain for active TRIP decreases, and the TRIP rate increases.

**Figure 4:**
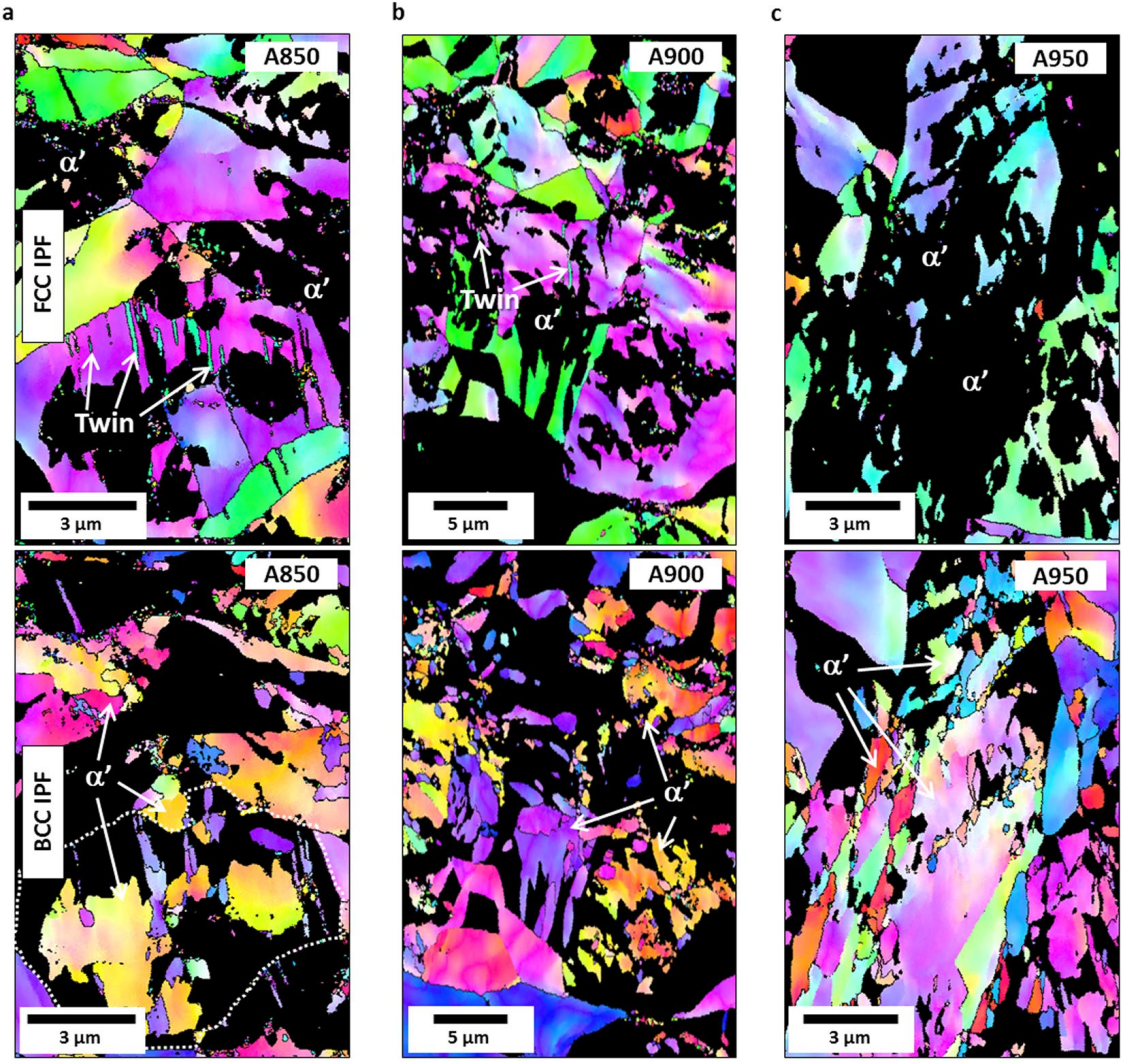
Deformation behavior with respect to TRIP and TWIP in Stage B of the annealed steels. (**a**,**b**) EBSD fcc and bcc IPF maps of the A850 and A900 steels at the true strain of 0.4. (**c**) EBSD fcc and bcc IPF maps of the A950 steel at the true strain of 0.2. In the A850 steel, a number of deformation twins and martensite are observed inside one austenite grain. In the A900 steel, only a few twins are found, while martensite is actively formed. Only martensite is formed without twins in the A950 steel.

In the Stage B of the three annealed steels, the TRIP occurs actively, but differently influences strain hardening rates and tensile properties. Since deformation mechanisms affect the transition in strain hardening behavior (Fig. 3(b)), the difference in deformation behavior needs to be carefully examined in the Stage B. Figure 4(a–c) shows IPF maps in the Stage B at the true strains of 0.4 (for the A850 and A900 steels) or 0.2 (for the A950 steel). In the A850 steel, a number of deformation twins and martensite are observed inside one austenite grain, as marked by a white dotted line in Fig. 4(a). When twins and martensite are formed simultaneously, the strain hardening rate increases, but its increased amount is small (Fig. 3(b)), which results in the steady stress flow behavior (Fig. 3(a)). In the A900 steel, only a few twins are found, while martensite is actively formed (Fig. 4(b)). This implies that the TRIP is activated as the austenite stability is decreased by the increased austenite grain size with increasing annealing temperature and by the decrease in C and Mn contents due to the increased austenite fraction. In the A950 steel, only martensite is formed without twins.

The stability of austenite is discussed in terms of alloying partitioning related with the fraction and grain size of austenite. In order to differentiate their contributions in the three annealing conditions, the Ms temperature is calculated by the following equations including effects of alloy composition and austenite grain size^38, 39^:

1 $${{\rm{Ms}}}_{0}(^\circ {\rm{C}})=539-423\,{\rm{C}}-30.4\,{\rm{Mn}}+30\,{\rm{Al}}$$

2 $${\rm{Ms}}(^\circ {\rm{C}})={{\rm{Ms}}}_{0}-{\rm{B}}({{V}_{\gamma }}^{-1/3})$$

where Ms_0_ shows the effect of alloy composition on Ms, and V_γ_ and B are the average volume of and geometry coefficient of austenite grain (475 μmK), respectively. C, Mn, and Al contents, grain size, and Ms temperature of austenite in the A850, A900, and A950 steels are listed in Table 1. When considering the effect of alloying composition only (Ms_0_), the difference in Ms_0_ temperature between the A850 and A950 steels is not large (14.5 °C). However, the Ms temperature is much lower by 105.5 °C in the A850 steel than in the A900 steel. This is because the decreased austenite grain size greatly raises the elastic strain energy required for martensitic transformation, and because the Ms temperature is inversely proportional to the grain size. This result indicates that the stability of austenite is mainly dependent on the grain size of austenite, although it is hard to differentiate their contributions experimentally.

**Table 1:** C, Mn, and Al contents, grain size, and Ms temperature of austenite in the A850, A900, and A950 steels.

Steel	C (wt.%)	Mn (wt.%)	Al (wt.%)	Grain Size (μm)	Ms_0_ (°C)	Ms (°C)
A850	0.39	10.81	4.17	1.7	170.5	−176.2
A900	0.46	10.23	4.22	2.1	160.9	−119.8
A950	0.52	9.62	4.29	2.6	156.0	−70.7

When the austenite stability is relatively high, like in the previous steel or A850 steel, the TRIP and TWIP mechanisms are working together, and the necking is retarded in the Stage C. Extremely high tensile ductility of 95% appears in the A850 steel, while the tensile strength remains high above 780 MPa. This maximization of tensile elongation is not desirable for the respect of strain hardening. When the TRIP is superior to the TWIP as the austenite stability decreases, the strain hardening is actively working. In the A950 steel whose transformation rate is too high, the elongation is seriously reduced. In the A900 steel, the high TRIP rate in the Stage B as well as the increased strain hardening rate (Fig. 3(b)) is observed. For example, the austenite transforms to the α′-martensite in the A850 steel (α′ volume fraction; 15%) from the stage B to the failure (during 0.3 strain), whereas it transforms in the A900 steel (α′ volume fraction; 30%) from the stage B to the failure (during 0.35 strain). The strain required for martensitic transformation is lower in the A900 steel than in the A850 steel, while the amount of transformation is larger, in spite of the less TWIP, thereby leading to the higher strain-hardening rate in the A900 steel. This result indicates that the strain hardening is more dependent on the TRIP than the TWIP. The rapid TRIP mechanism in the Stage B is closely related with the deterioration of austenite stability, and thus the A900 steel shows the higher tensile strength and lower elongation than the A850 steel. When considering the high strain hardening without plastic instability, the austenite stability of the A900 steel is adequately optimized for structural applications.

### Comparison of present duplex steels with conventional highly-deformable high-strength steels

**Figure 5:**
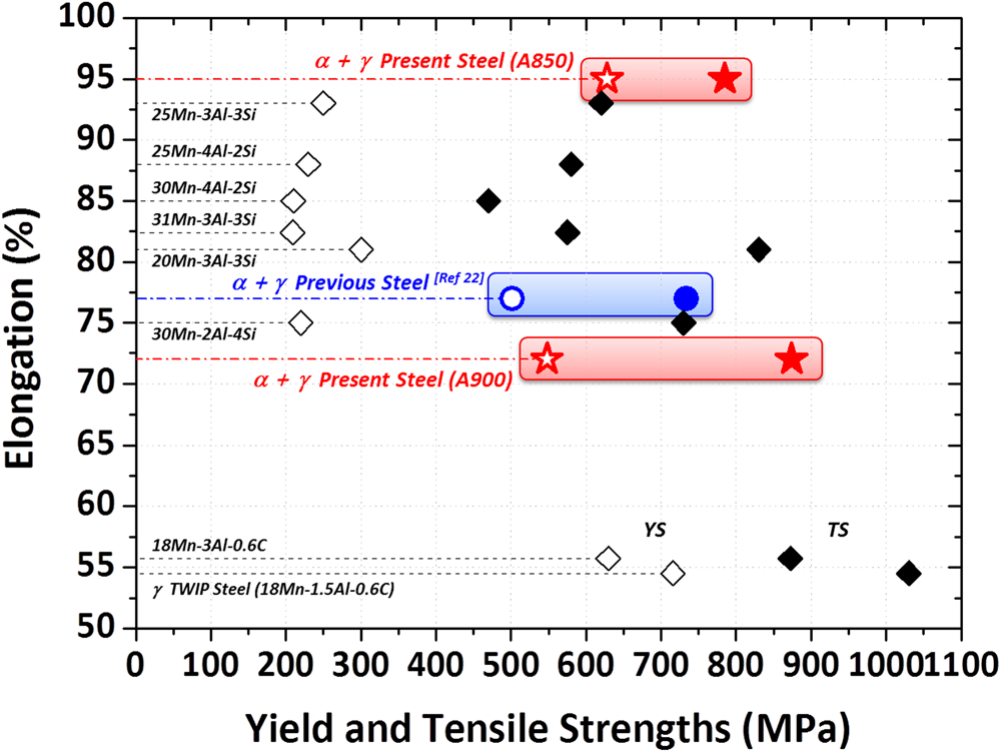
Comparison of room-temperature tensile properties of the present Fe-0.3C-9Mn-5Al duplex lightweight steels with conventional highly-deformable TWIP steels. The typical TWIP steels whose composition is Fe-18Mn-(1.5,3)Al-0.6C show an excellent tensile strength (870~1050 MPa) with relatively low elongation (about 55%)^36^. The TWIP steels containing high-Mn contents of 20~31 wt.% with Si addition show the high elongation of 75~93%, which is comparable to those of the present lightweight steels, but their yield strength is relatively low (210~300 MPa)^37, 38^. Considering the negative effects of high-Mn addition, the present lightweight steels containing relatively lower Mn content (9 wt.%) have merits of similar or more excellent tensile properties as well as low alloying costs and lightweight effects (about 8% weight reduction).

Recently, high-Mn TWIP steels have been nominated as promising automotive steels having excellent combination of strength and ductility^40–45^. Figure 5 shows the comparison of the present lightweight steels with the previous steel and recently developed Fe-Mn-Al or Fe-Mn-Al-Si TWIP steels showing impressive tensile ductility over 50%^43–45^. The typical TWIP steels whose composition is Fe-18Mn-(1.5,3)Al-0.6C show an excellent tensile strength (870~1050 MPa) with relatively low elongation (about 55%)^43^. When the Mn content increases to 20~31 wt.% with Si addition, the elongation increases to 75~93%, which is comparable to those of the present lightweight steels, but the yield strength decreases down to 210~300 MPa^44, 45^. These austenitic microstructures show rather low yield strength because of their inherent characteristics and grain coarsening^46, 47^. In this respect, the duplex microstructure has been regarded as a desirable concept because of its advantages of high yield strength. In addition, the high Mn content in TWIP steels often causes problems such as reduced productivity due to temperature drop of the molten steel during steel-making, nozzle blocking during continuous casting, cracking during hot rolling, and easy surface oxidation of rolled steel products^48^. Considering these negative effects of high-Mn addition, the present lightweight steels containing relatively lower Mn content (9 wt.%) have merits of similar or more excellent tensile properties as well as low alloying costs and lightweight effects (about 8% weight reduction).

## Conclusions

In conclusion, when considering the strength and elongation simultaneously, the austenite having an appropriate stability is desirable. In the duplex microstructures, the alloying partitioning due to variation in austenite fraction should be importantly considered. As aforementioned in the section of alloy design concept, the indirect decrease in austenite stability due to the austenite fraction increased by the increased Mn and decreased Al contents acts as an effective strategy of alloy design. Since the austenite greatly affects tensile properties, depending on its stability, it does not sufficiently contribute the ductility improvement in the case of the A950 steel having very low stability. In order to obtain the best combination of strength and ductility, the formation of austenite having an appropriate stability is essentially needed, and can be achieved when about 55 vol.% of fine austenite (size; about 2 μm) is distributed, like in the A850 and A900 steels. The A850 steel shows excellent elongation (95%) as well as high tensile strength (785 MPa), which are superior to those of the previous steel^22^. This excellent combination of strength and ductility is basically attributed to multiple-stage deformation mechanisms, *i.e*., simultaneous formation of deformation-induced martensite and deformation twin and additional plasticity by twinning. However, this is not desirable for the issue of high strain hardening rate generally required in deformability, fracture resistance, and energy absorption capability. With respect to this issue, the TRIP mechanism prevails more actively in the A900 steel than the TWIP mechanism as the austenite stability is somewhat lower than that of the A850 steel. The A900 steel also shows a high strain hardening, together with an excellent combination of strength and ductility (874 MPa and 72%, respectively). Since the A900 steel shows outstanding tensile properties as well as reduced density, they give a promise for automotive applications to highly-deformable high-strength sheets and structural reinforcement components requiring high stiffness.

## Methods

### Fabrication of lightweight steels

The lightweight steel, whose chemical composition was Fe-0.3C-9Mn-5Al-(<0.02)(P + S) (wt.%), was fabricated by a vacuum induction melting method. Effects of Al addition on weight reduction are attributed to lattice expansion and low atomic weight of substitutional solution^37^. The addition of 1 wt.% Al leads to a 1.5% weight reduction in comparison with conventional steels. 60-mm-thick plates homogenized at 1200 °C for 1 hour were hot-rolled between 1100 °C and 900 °C, and were cooled in a furnace from 650 °C after holding at this temperature for 1 hour to simulate a coiling procedure. 3-mm-thick hot-rolled steel sheets were rolled again at room temperature to produce 1-mm-thick steel sheets. The sheets were annealed at 850, 900, and 950 °C for 5 min in a continuous annealing simulator (model; CAS-AY-II, Ulvac-RIKO, Inc., Japan) to form (ferrite + austenite) duplex microstructures, were cooled to 400 °C at a rate of -14 °C s^−1^ to avoid the decomposition of austenite, and were air-cooled. Considering the requirement of short-time annealing in the practical continuous annealing line, the annealing time was determined to be 5 min.

### Microstructural analysis

The annealed steel sheets were mechanically polished and then electro-polished at room temperature in a solution of CH_3_COOH (90%) and HClO_4_ (10%) at an operating voltage of 32 V for the EBSD observation of microstructures of longitudinal-short-transverse (L-S) plane. The EBSD analysis (step size; 50 nm) was performed by a field emission scanning electron microscope (FE-SEM, Quanta 3D FEG, FEI Company, USA), and the data were interpreted by orientation imaging microscopy (OIM) analysis software provided by TexSEM Laboratories, Inc. Phases present in the annealed steels were identified by X-ray diffraction (XRD, Cu K_α_ radiation, scan rate; 2 deg min^−1^, scan step size; 0.02 deg). Their volume fractions were measured using XRD analysis^49^. Integrated intensities of (200)_α_ and (211)_α_ peaks and (220)_γ_ and (311)_γ_ peaks were used for this XRD method. Chemical compositions in austenite were analyzed by electron probe micro-analysis (EPMA) in an EPMA microprobe (model; JXA 8530F microprobe, JEOL, Japan). Because of the difficulty in precise measurement of C, the C content was measured by the XRD method^50^.

### Tensile test

Plate-type tensile specimens (gage length; 25 mm, gage width; 6 mm, gage thickness; 1 mm) were prepared in the longitudinal direction. They were tested at room temperature at a strain rate of 10^−3^ s^−1^ by a universal testing machine of 100 kN capacity (model; Instron 8801, Instron Corp., Canton, MA, USA), in accordance with the ASTM E 8/E 8 M standard specification. The tensile tests were conducted three times for each datum point.

### Data Availability

The data that support the findings of this study are available from the corresponding author upon reasonable request.
